# Supplementary material for: “I’m Torn”: Qualitative Analysis of Dental Practitioner-Perceived Barriers, Facilitators, and Solutions to HPV Vaccine Promotion
Source: Healthcare (Basel). 2024 Apr 3;12(7):780. doi: 10.3390/healthcare12070780 (PMC11011463; doi:10.3390/healthcare12070780)
Supplement: Supplementary file 1 [file healthcare-12-00780-s001.zip › HPV Dental_Practitioner Interview_Interview Guide_v1.0.pdf]

# HPV Dental – Practitioner Interviews

Interview Guide

---

**Interview Questions:**

Root 1: For routine visits, who on your care team (meaning you and the [dentist/hygienist/dental therapist] you work with) usually makes preventive oral health recommendations to patients? By oral health recommendations, I mean things like regular brushing and flossing, tobacco cessation, or avoiding sugary drinks.

- Is this standard throughout your clinic or does it vary by care team or dyad?
- Does your care team's approach differ by preventive health topic? Please explain.
- How does your care team decide who initiates different oral health conversations?
- How do you communicate within your care team about these conversations during a visit?
- Does your care team's approach differ based on the age of the patient or if a parent is present?

Root 2: Have you ever talked to a patient or parent about HPV and/or the HPV vaccine?

[If no] Can you tell me about a time you talked to an adolescent, a parent, or a young adult patient about a different preventive oral health topic? Other practitioners have used tobacco cessation as an example.

- How did you start that conversation? How did the patient respond?
- When you give a recommendation, do you ever ask a patient or parent how likely they are to follow through on the recommendation? Do you ever talk about barriers or try to find solutions?
- In general, how does your approach to communication change when talking to a younger adolescent, say an 11- or 12-year-old? What about teens, a 16- or 17-year-old? If a parent is present? At what age do parents usually stop attending routine visits? Have you ever made a recommendation that a parent and child react to differently? How do you approach that conversation?
- How do you prioritize patient needs during a visit? If a patient presents with complex oral health needs or is at high risk, do you approach recommendations differently? If a patient has complex social needs, do you approach recommendations differently?
- How do you think about cultural differences in your practice? Do you ever think about your own biases when making recommendations?

Now I'd like to talk more specifically about HPV.

[If yes] Did you talk about HPV, the HPV vaccine, or both? Tell me about the experience.

- How did you start that conversation? How did the patient respond?

- When you give a recommendation, do you ever ask a patient or parent how likely they are to follow through on the recommendation? Do you ever talk about barriers or try to find solutions?
- In general, how does your approach to communication change when talking to younger adolescents? Teens? If a parent is present? At what age do parents usually stop attending routine visits? Have you ever made a recommendation that a parent and child react to differently? How do you approach that conversation?
- How do you prioritize patient needs during a visit? If a patient presents with complex oral health needs or is at high risk, do you approach recommendations differently? If a patient has complex social needs, do you approach recommendations differently?
- How do you think about cultural differences in your practice? Do you ever think about your own biases when making recommendations?

<u>Root 3</u>: What are your thoughts on dental practitioners recommending the HPV vaccine during routine dental visits?

- Do you think other practitioners feel the same way? Why or why not?
- If your clinic was asked to start recommending the HPV vaccine to patients, would you likely be the person to make the recommendation or the [dentist/hygienist/therapist] you work with? Is that the same for other dentists and hygienists in your clinic?
- If your care team was asked to make this recommendation, what concerns would you have? What about others in your clinic? [priority] What would you need to feel ready to make an HPV-V recommendation to a patient?
- In the past when you've been asked to make a new recommendation or follow a new guideline, what support was most helpful? What support did you wish you had?

<u>Root 4</u>: If your care team was asked to recommend the HPV vaccine to patients, what information might you need to know beforehand?

- Would it be helpful to learn more <u>about HPV</u>? What information would you like to learn?
    - For example, would it be helpful to learn more about the clinical course of HPV? … cancer risk of individuals infected with HPV? …how HPV is transmitted? Or the epidemiology of HPV and oral cancer?
- Would it be helpful to learn more <u>about the HPV vaccine</u>? What information would you like to learn?
    - For example, would it be helpful to learn more about eligibility for the vaccine? …how many doses are recommended? …the type of vaccine? …the manufacturer? …side effects? …efficacy of the vaccine?
- Would it be helpful to learn more <u>about vaccine logistics</u>, such as how or where patients can get the vaccine? Or whether patients have to pay for the vaccine?
- [priority] How would you like to learn this information? Can you tell me about a time you received education on a new topic that was really useful? What did you like? What didn't you like?

<u>Root 5</u>: How do you think your patients would respond to you recommending the HPV vaccine?

- Would you worry about patients' or parents' responses to recommending the vaccine? Would you worry about parents of young adolescent patients' response to recommending the vaccine?
- What concerns might they have about the vaccine? Do you think patients or parents have opinions about this vaccine that are different than their opinions about other vaccines? Do you think patients or parents are hesitant about routine vaccines? If a patient or parent raises one of these concerns, how would you respond?
- How confident would you feel in making a strong recommendation to patients that they get this vaccine? What makes you say that? What would help make you feel more confident?
- How confident would you feel in answering questions a patient or parent might have about HPV or the HPV vaccine? What would help make you feel more confident?
- How confident would you feel in making a strong recommendation if a patient or parent raises concerns about HPV, the HPV vaccine, or vaccines generally? What would help make you feel more confident?
- Do you think you could get in the habit of recommending the vaccine to all your patients who are eligible? What might get in the way?
- If you don't have much time in a visit, could you still make this recommendation? What about if a patient has other complex needs?
- What other things might get in the way of making this recommendation?

Root 6: What support would be helpful from your clinic or the health system if you were asked to make a HPV vaccination recommendation to patients?

- What would you like to see in a training about HPV and HPV vaccine?
- Tell me about a time you received quality training that influenced how you make oral health recommendations to patients. What about this training was particularly useful? How was this training structured?
    - How confident are you communicating with patients who seem resistant to change? What communication skills would you like to improve? Have you ever done any training on communication approaches?
        - [If yes] Was it useful? Why?
        - [If no] Would that be useful? Why?
    - Have you ever done any role-play with patient in a training? How would you feel about that type of learning?
    - From your experience, is there anything specific we should avoid in preparing a training of this sort?
- Have you ever used scripted messages or talking points to deliver a recommendation or address patient concerns?.
    - [If yes] What did you think of it? How did you use these during your visit? What did you like? What didn't you like? How could this approach be changed to be more useful?
    - Was your experience with scripted messages more or less useful compared to other training or professional development you have received? Why?
    - [If no] Do you think it would be useful? What would make it more useful?
    - What messages (content) would be helpful to have provided in talking points?
    - Our team is also looking at the literature to inform these talking points. What would you say if a parent or young adult patient told you…
        - They were not worried about HPV-related cancers?
        - They were not concerned about genital warts?

        - They were not concerned about the possibility of spreading HPV to others?
        - They want to avoid pain at the injection site?
        - They are worried about other vaccine side effects?
        - They do not follow provider recommendations?
    - Would it be useful to have scripted questions to ask patients or parents about how likely they are to get vaccinated? Would it be helpful to have scripted questions to ask the parent or patient about their concerns? Their motivations for getting the vaccine?
- If you were given scripted messages to help you respond to parent or patient questions, what would make these useful?

Root 7: Do you have any other concerns about recommending HPV vaccination to your patients? Is there anything else you'd like to share?
